# Patient profiles as an aim to optimize selection in the second line setting: the role of aflibercept

**DOI:** 10.1007/s12094-021-02568-y

**Published:** 2021-02-25

**Authors:** B. González Astorga, F. Salvà Ballabrera, E. Aranda Aguilar, E. Élez Fernández, P. García-Alfonso, E. González Flores, R. Vera García, A. Fernández Montes, A. M. López Muñoz, A. Salud Salvia

**Affiliations:** 1 Hospital Universitario Clínico San Cecilio, Granada, Spain; 2 Hospital Universitari Vall d’Hebron, Barcelona, Spain; 3 Hospital Universitario Reina Sofía, Córdoba, Spain; 4 Hospital General Universitario Gregorio Marañón, Madrid, Spain; 5 Hospital Universitario Virgen de Las Nieves, Granada, Spain; 6 Complejo Hospitalario de Navarra, Navarra, Spain; 7 Complexo Hospitalario de Ourense, Ourense, Spain; 8 Hospital Universitario de Burgos, Burgos, Spain; 9 Hospital Universitari Arnau de Vilanova, Lleida, Spain

**Keywords:** Colorectal cancer, Metastatic, Treatment, Aflibercept, Patient selection, Patient profile, RAS, BRAF, Mutation

## Abstract

Colorectal cancer is the second leading cause of cancer-related death worldwide. For metastatic colorectal cancer (mCRC) patients, it is recommended, as first-line treatment, chemotherapy (CT) based on doublet cytotoxic combinations of fluorouracil, leucovorin, and irinotecan (FOLFIRI) and fluorouracil, leucovorin, and oxaliplatin (FOLFOX). In addition to CT, biological (targeted agents) are indicated in the first-line treatment, unless contraindicated. In this context, most of mCRC patients are likely to progress and to change from first line to second line treatment when they develop resistance to first-line treatment options. It is in this second line setting where Aflibercept offers an alternative and effective therapeutic option, thought its specific mechanism of action for different patient’s profile: RAS mutant, RAS wild-type (wt), BRAF mutant, potentially resectable and elderly patients. In this paper, a panel of experienced oncologists specialized in the management of mCRC experts have reviewed and selected scientific evidence focused on Aflibercept as an alternative treatment.

## Background

Colorectal cancer (CRC) is the second leading cause of cancer-related death worldwide, with an incidence that will increase from 1.8 million new cases in 2018 to 2.5 million in 2035 [1, 2]. Despite the great efforts and numerous screening programs aimed to reduce CRC incidence, approximately 20% of patients will present with metastatic spread at the time of diagnosis, and a significant proportion of previously treated patients have disease recurrence typically involving the liver or lungs.

Moreover, it is well known that colorectal cancer (CRC) is a complex and heterogeneous disease. In the recent years, a better understanding of the underlying biology associated to this disease has led to incorporate biologically targeted drugs and optimize the therapeutic strategy based on the genomic characteristics of the tumor. Today, the molecular determination of *RAS*, *BRAF* and microsatellite instability (MSI), and clinical biomarkers such as primary tumor location are essential to properly select patients who are candidates for certain biological treatments [3–5]. In spite of these advances, the 5-year overall survival of these patients with advanced disease is still less than 15% [4].

Currently, European Society for Medical Oncology (ESMO) guidelines [6] and the Pan-Asia adaptation [7] recommend chemotherapy (CT) based on doublet cytotoxic combinations of fluorouracil, leucovorin, and irinotecan (FOLFIRI) and fluorouracil, leucovorin, and oxaliplatin (FOLFOX) as first-line treatment for mCRC patients. Furthermore, biological (targeted agents) are indicated in the first-line treatment of most patients unless contraindicated. For example, whereas the use of EGFR antibody therapy is very common in patients with mCRC and left-sided RAS wild-type (wt), right-sided RAS wt tumors might be better treated with chemotherapy alone or chemotherapy plus bevacizumab—except maybe if the goal is tumor size reduction as the overall response rates (ORRs) were higher, but not progression free survival (PFS) and overall survival (OS).

However, most patients will progress and will need change from first line to second line treatment, among other, when they show resistance to first-line treatment options [8, 9]**.** It is in this second line setting where Aflibercept, a vascular endothelial growth (VEGF) targeted agent, has shown precise biological activity to treat advanced CCR. However, this treatment is not the only one in this setting. Depending on previous lines and patient characteristics, oncologists must decide the most appropriate strategy of treatment. In this review, we share scientific evidence of Aflibercept used as an alternative treatment for mCRC patients with different profile that progress under first-line treatment.

The publications and articles that make up this review have been selected based on a panel of experts. Five advisory boards have been carried out in Spain during April and May 2020. During the meetings, the experts’ opinions were mainly based on scientific evidence coming from clinical trials and Ad-Hoc studies focused on Aflibercept as an alternative treatment. The expert panel that authored this review is represented by different oncologists specialized in the management of mCRC that have been selected on scientific merits and daily clinical experience of managing mCRC patients.

## Aflibercept presents a unique differential mechanism of action

Angiogenesis is the complex process of formation of new blood vessels due to over expression of VEGF (vascular endothelial growth factor) which plays a critical role in the growth and development of all solid tumor types. The advancement in understanding of tumor angiogenesis and VEGF has resulted in the development of various agents capable of targeting VEGF for the treatment of cancer [10]. In a similar way, as observed in other solid tumors, angiogenesis has been shown to play an important role in CRC development and progression.

Aflibercept’s specific mechanism of action functions blocking pro-angiogenetic VEGFs, including VEGF-A, VEGF-B, and placental growth factor (PlGF-1 and-2), thereby preventing VEGFs and PlGF from binding to native VEGF receptors [8, 11]. In contrast to bevacizumab, which only binds to VEGF-A by forming multimeric complexes [12], or with ramucirumab, which specifically inhibit VEGFR-2, aflibercept monomerically binds to all VEGF-A isoforms, in addition to VEGF-B, and PlGF [13], thus showing a unique mechanism of action.

These differences in the mechanism of action may be relevant to identify potential biomarkers to detect potential resistance to antiangiogenic drugs.

In tumors, as it occurs with the gene encoding VEGF, PlGF gene may also be increased after administration of cytotoxic anticancer treatments. The circulating levels of PlGF protein can also markedly increase during anti-VEGF therapy, which suggests that PlGF might contribute to the compensatory mechanism of drug resistance. In this line, various studies show a trend in the resistance for first-line mCRC treatment [8, 14] that leads to progression of the disease.

A recent expert opinion article [8] highlights that PlGF is the most relevant mediator of resistance to bevacizumab and that aflibercept potentially offers a more complete inhibition of angiogenesis as it is the only mCRC treatment that blocks specifically PlGF, VEGF-A and VEGF-B activation.

Despite all the evidence mentioned above, these biomarkers are not used in routine clinical practice and further studies are needed.

From the beginning, aflibercept has demonstrated the ability to block tumor-associated angiogenesis. In 2002, first preclinical study demonstrated already aflibercept’s ability of inhibiting VEGF-induced phosphorylation in human umbilical vein endothelial cells and inhibition of proliferation in fibroblasts [15].

In 2012 it was conducted the phase III VELOUR study [16], that emerged from the uncovered need of providing an alternative treatment for those mCRC patients resistant to or progressive after an oxaliplatin-containing regimen. Moreover, until 2012, none biologic therapy had demonstrated to provide statistically significant survival benefit in this type of patients.

VELOUR trial [16] is a phase III, multinational, prospective, double-bind study that aimed to assess the efficacy and safety of aflibercept in combination with FOLFIRI versus placebo plus FOLFIRI in mCRC patients with progression on a prior oxaliplatin-containing regimen. A total sample of 1226 patients were randomized in two study arms, FOLFIRI/placebo and FOLFIRI/aflibercept, which was administered at maximum dose. By contrast to other second-line studies [17], VELOUR patients were not selected for the timing of their progression and could have been treated previously with bevacizumab [16].

According to VELOUR results [16], OS median survival was 13.50 months in FOLFIRI/aflibercept versus 12.06 months in placebo. It is also worth highlighting that two-year survival rates were 28.0% in the experimental arm versus 18.7% in placebo, showing that the addition of aflibercept to FOLFIRI produced a continuous and persistent improvement over time in OS. Further, in 2016, an article based on VELOUR outcomes [18] reported that addition of aflibercept to FOLFIRI showed a continued and persistent improvement over time in OS in patients. Furthermore, aflibercept also demonstrated to significantly improve median PFS being 6.90 months in FOLFIRI/aflibercept versus 4.67 in placebo (*p* < 0.0001). Concerning response rate (RR), it was 19.8% in aflibercept treatment versus 11.1% in placebo (*p* < 0.0001). Thus, VELOUR demonstrated that treating patients with progression on a prior oxaliplatin-containing regimen with aflibercept, leaded to statistically significant improvement in overall response rate (ORR), overall survival (OS) and progression free survival (PSF).

In 2020, it was published another post-hoc analysis of VELOUR trial [11] that concluded that aflibercept retains its activity regardless of baseline VEGF-A and PlGF levels and may be an effective alternative treatment for patients with bevacizumab-induced resistance.

## Aflibercept patient profile

Selecting the most optimal treatment strategy (first-line subsequent) in each patient profile, using all available drugs in appropriate way as a part of a “continuum of care”, has great impact on patients’ outcomes. For this reason, different follow-up VELOUR sub-analysis and independent studies were carried out in order to assess role of aflibercept in different patients’ profile.

### RAS mutant patients

Concerning to RAS mutant patients, taking as a reference ESMO guidelines [6], aflibercept is postulated as a second-line recommended in patients who had received prior oxaliplatin-based treatment with or without bevacizumab.

The recently published ITACa study [19] compared two different sequences of treatment for mCRC. Despite the study itself should be interpreted with caution due to the methodological design and biases, results also support that second line treatment could be an ideal setting for angiogenesis inhibition.

In second line treatment context, there are available several phase III studies that explore the role of antiangiogenic treatment with different drugs: Aflibercept (VELOUR trial) [16], Bevacizumab (TML) [17], E3200 [20] and Ramucirumab (RAISE) [21]. It is worth mentioning that population included differs between these studies, as well as the treatment received in first line. As consequence, these factors are taken into account when deciding the most suitable treatment for each patient. Thus, as The Eastern Cooperative Oncology Group Study E3200 [20] included patients after progression to a previous irinotecan-based regimen and, since patients’ profile are different from the ones discussed in the present review, this study will not be developed in greater detail.

If it is used exclusively CT (chemotherapy) with FOLFOX scheme as first-line treatment, the most recommended scheme in second-line would be FOLFIRI-aflibercept, as previously commented on the ITACa study [19] and the E3200 Study with bevacizumab (after having used irinotecan) [20].

In the case of using the FOLFOX-bevacizumab scheme in the first line, there are several possible options:

- According to inclusion criteria of phase III ML18147 trial [17], excluded patients who received less than 3 months of bevacizumab in the first-line setting or who developed progression more than 3 months after the last treatment with bevacizumab. Randomization was stratified depending on first-line chemotherapy scheme (irinotecan-based vs oxaliplatin-based), first-line PFS (≤ 9 months vs > 9 months), time from last bevacizumab dose (≤ 42 days vs  > 42 days), and ECOG performance status (0 or 1 vs 2).
  After randomization, participants were assigned to second-line CT either with bevacizumab or without bevacizumab. According to results, median OS was 11.2 versus 9.8 months (HR, 0.81, 95% CI, 0.69–0.94), unstratified log-rank test (*p* = 0.0062), and median PFS was 5.7 versus 4.1 months, in CT plus bevacizumab compared to CT alone, respectively. Regarding treatment response (RR), similar results were observed in both groups, being achieved by 6% of the experimental arm versus 4% of patients treated with CT alone (Table 1).

**Table 1:** OS, PFS and RR results according to antiangiogenic agents in second-line combination therapy

	Aflibercept	Bevacizumab	Ramuricumab
	VELOUR	TML ML18147	RAISE
OS (months)	13.5 vs 12.06	11.2 vs 9.8	13.3 vs 11.7
PFS (months)	6.9 vs 4.7	5.7 vs 4.1	5.7 vs 4.5
RR (%)	19% vs 11.1%	6% vs 4%	13% vs 13%
  In conclusion, ML18147 trial [17] provided evidence for a continuation of bevacizumab beyond progression of disease on first-line therapy. Prespecified subgroup analyses of OS in the intention-to-treat (ITT) population were generally consistent with the primary findings; although the subgroup of patients that seems to obtain the greatest benefit are those with first-line treatment (PFS > 9 months, HR 0.73 (0.58–0.92)).
- By contrast to ML18147 Study [17], VELOUR Study [16] included patients with rapid progression at first-line treatment (PFS < 3 months), those whose progression was > 3 months after the last dose of Bevacizumab, and patients who relapsed after 6 following months after the end of the Adjuvant rapid relapsers (ARR). With focus on the subgroup of patients previously treated with bevacizumab, the post-hoc analyzes [18] have demonstrated that if patients with rapid progression to adjuvant are retired (ITT minus ARR (ITT-ARR) population), who are the population with the worst prognosis, the magnitude of the benefit increases with a median OS of 13.80 months vs 11.66 months (absolute increase 2.14 months). This analysis also shows that, in the ITT-ARR population, there is benefit regardless of the time of first line progression (< 3 months,  ≥ 3 to < 6 months,  ≥ 6 to < 9 months, and ≥ 9 months). In the ITT-ARR population that had previously received bevacizumab, this benefit was also observed regardless of the time of progression of the first-line disease [ITT-ARR prior to bevacizumab  ≥ 9 months HR 0.73 and absolute increase in OS of 3.97 months (17.64 m vs 13.67 m)].
- In a phase III clinical trial, the RAISE Study [21], it was evaluated the efficacy and safety of the antiangiogenic ramucirumab, compared to placebo, in combination with second-line FOLFIRI in mCRC patients who had previously received bevacizumab. The results reported a median OS of 13.3 months versus 11.7 months and a median PFS of 5.7 months versus 4.5 months, being the data in treatment with ramucirumab superior to placebo. Regarding RR, the proportion of patients who achieved an objective response was similar, being 13% for both groups (Table 1). Though, this option is not approved in the Spanish Healthcare System.
  Overall survival stratified hazard ratios (HRs) are similar in the RAISE (0.84) [21], TML (0.83) [17], and VELOUR [16] trials (0.82 in the total population and 0.86 in the subpopulation of patients who had received first-line bevacizumab). However, it is noteworthy that cross-trial comparisons are limited by differences in design, study populations, efficacy and safety measures, and analysis methods. Regardless their differences, all three trials support the hypothesis that inhibition of tumor angiogenesis beyond initial disease progression is an effective treatment strategy.

### RAS wild-type (wt) patients

In 2017, it was conducted a study [22] that acquired tumor samples from phase III VELOUR trial [16] for biomarker analyses. Results reported that the best OS, PFS and RR corresponded to wild-type patients, thus being patients without mutation in KRASex2 gene the ones that reached higher median OS (16 months) and RR (28.6%).

The right sequence of second-line treatment for RAS wild-type patients has yet to be defined. In 2019, Galvano et al. [23] conducted a meta-analysis of anti-EGFR and anti-VEGF therapies in second-line mCRC patients by analyzing efficacy and safety data from phase III clinical trials. For the first time, this meta-analysis showed an improved trend towards OS and disease control rate (DCR) for anti-VEGF treatment in second line, in contrast to anti-EGFR therapy. Additionally, to compare the selected trials, they made a quality analysis from which VELOUR study was the only one that met all quality criteria.

Both EGFR antibodies, cetuximab and panitumumab, have demonstrated to increase RR and PFS, but not OS when combining with irinotecan-containing therapy in the second-line treatment setting [24–26]. A similar relative benefit has been confirmed in a randomized clinical trial [27] when cetuximab (and panitumumab) is used in later lines compared with second line. Assuming that exposure to all drugs increases survival, one strategy would be to use aflibercept as the second line and reserve EGFR antibodies for the third line treatment.

Likewise, this profile of patients will preferably receive a combination of chemotherapy (CT) plus anti-EGFR as first line treatment. It is well established that EGFR inhibitors are only effective in RAS wt patients [28, 29] and meta-analysis of first-line head-to-head studies with biologic agents in RAS wt patients support their use versus bevacizumab [30, 31].This seems certain in patients with left-sided tumors according to the meta-analysis results of the location of primary tumor [32, 33] that confirms the anti-EGFR prognostic and predictive role and support that patients with left-sided wt RAS mCRC should preferably be treated with an anti-EGFR antibody in first-line. However, these predictive results should be interpreted with caution due to the retrospective nature of the analysis. Hence, a combination of FOLFOX with either cetuximab or panitumumab is often used in the first-line setting in patients with wt RAS mCRC.

With regards to VELOUR Study [16], RAS wt population treated in the first line with a combination of FOLFOX and anti-EGFR is underrepresented. For this reason, the evidence that supports the best sequence in the second line should be searched in other types of studies, such as those based on real world evidence.

Recently in Spain, a retrospective observational study [34] was conducted to assess the efficacy and safety data of FOLFIRI/aflibercept in wt RAS mCRC patients after progression to standard CT + anti-EGFR treatment, a sample of 120 patients were included in the study. The results were quite similar to those derived from VELOUR [16]; median OS was 14.5 months and PFS was 6.9 months. Besides, it was obtained an outstanding ORR percentage (33%). Based on corresponding results, it was suggested that FOLFIRI/aflibercept after first-line treatment with anti-EGFR is an appropriated option for wt RAS mCRC.

Additionally, ESMO Guidelines [6] indicate that the use of anti-EGFR treatment is not recommended if it has been previously used.

### BRAF mutant patients

BRAF V600E mutations, found in about 8–12% of colorectal cancer patients define a particular subtype of mCRC, characterized by a discouraging prognosis.

In the absence of prospective phase 3 studies in this population, The ESMO guidelines (3) suggest first-line treatment in BRAF mutated (BRAF-mt) patients based on triplet chemotherapy with bevacizumab (if it is not contraindicated), based of post-hoc analysis of 28 patients included in Tribe trial [35]. In spite of the evidence level remains low, this strategy is well accepted because it offers an aggressive upfront treatment to treat patients with a particularly aggressive disease who are rarely able to receive a second-line treatment. Recently, a metanalysis with five randomized control trials involving triplet strategy in mCRC, showed no overall survival benefit of FOLFOXIRI plus bevacizumab among the 9% of patients with *BRAF*-mutant tumors (HR = 1.11, 95% CI = 0.75–1.73) [36].

Different post-hoc analyses of randomized trials in second line scenario suggest that anti-angiogenic agents might be of interest in BRAF-mt mCRC patients. However, prospective trials comparing an aggressive chemotherapy alone or in combination with an anti-angiogenic therapy in this this setting are still awaited. Wirapati [37] reported results related to 36 BRAF mutated patients included in VELOUR trial: median OS for FOLFIRI/aflibercept patients almost doubled median OS of FOLFIRI/placebo patients, being 10.3 and 5.5 months, respectively. Further, PFS reported a median of 2.2 months in FOLFIRI/placebo patients and 5.5 in those treated with FOLFIRI/aflibercept. Concerning RAISE study [21], a sample of 41 patients were BRAF mutant [38]. For this profile of patients, results reported a median OS of 9 months and a median PFS of 5.7 months.

Recently, in the BEACON phase III study [39], it was evaluated encorafenib + cetuximab plus or minus binimetinib versus either irinotecan/cetuximab or FOLFIRI/cetuximab, as controls, in patients with BRAFV600E mCRC whose disease had progressed after 1 or 2 prior regimens in the mCRC setting. According to results, median OS was 9.0 months in the triplet-treated group vs 5.4 months in the control group. RR was 26% in triplet therapy group vs 2% in those patients treated with placebo. Regarding doublet therapy results, median OS obtained for treated patients was 8.4 vs 1.5 months in the placebo group. RR was 20% in doublet group. It was concluded that combination of encorafenib, binimetinib, and cetuximab or combination of encorafenib and cetuximab, in comparison with current standard therapy, resulted in a significant and clinically relevant benefit with respect to overall survival and objective response rate.

Moreover, the second line treatment can be conditioned by data from the ANCHOR phase 2 study [40], which has recently reported up to a 50% response rate with the combination encorafenib, cetuximab binimetinib in first line. Further results of this trial are still awaited, and these results could condition following lines.

The place of each therapeutic combinations described and the way to sequence these new options remains an open question today. Further investigations are therefore justified. Promoting the enrollment of BRAF-mt mCRC patients in clinical trials is urgently needed.

### Potentially resectable patients

According to Pan-Asian adapted ESMO consensus guidelines [7], it is indicated that any mCRC patients with limited liver and/or lung metastases should be considered a candidate for potential secondary resection. Resection rates have been shown to be correlated with response to systemic therapy in colorectal cancer liver metastases (CLM) [41].With regards to CLM, Adam and colleagues [42] reported that 49% of patients are still alive at 5 years, regardless of whether surgery was after 1L or 2L, with a similar response not be denied after the failure of first-line chemotherapy. For this reason, response rate should be considered as an important endpoint in those patients with potentially resectable disease.

Taking this into account, it should be noted that the VELOUR study [16] achieved a response rate of up to 19% with FOLFIRI-Aflibercept in the second line, being one of the highest RR reported in this scenario.

In 2019, a retrospective, observational study conducted by Muñoz et al. [43] collected and analyzed data from 39 patients from 32 Spanish hospitals who underwent metastases resection after FOLFIRI/aflibercept in a real-life setting. Results showed that, after surgery, median OS after surgery was 35.2 months and median PFS was 10 months. On the report of these results, authors concluded that resection of metastases after FOLFIRI/aflibercept in oxaliplatin-refractory patients is safe and feasible with encouraging results in OS. In a similar way, another study [44] reported that 18% of patients who underwent secondary metastatic resection had received aflibercept with a median OS of 51 months.

In conclusion, CLM resection following second-line preoperative chemotherapy (PCT) [42], after onco-surgical favorable selection, could bring similar OS compared to what observed after first-line. For initially unresectable patients, OS or PFS is comparable between first- and second-line PCT. Surgery should not be denied after the failure of first-line chemotherapy.

### Elderly patient

Although patients ≥ 65 years of age represent the majority of patients with CRC, they are often underrepresented in clinical trials of mCRC. This affects the oncologists, thus, in the near future, they will have to confront increasing numbers of older CRC patients (56). The global proportion of older people (> 60 years) will increase from 11.7% in 2013 to 21.1% by 2050. In Europe, the proportion of elderly people (≥ 65 years) will reach 28% by 2050 [45]. Despite the underrepresentation of the elderly population in randomized clinical trials, adding biological treatments to chemotherapy significantly prolongs OS in this population, as demonstrated by the meta-analysis with 8488 patients reported by Zhao et al. [46]

In 2018, a subset analysis of the VELOUR study [47] was undertaken to investigate efficacy and safety of FOLFIRI/aflibercept versus FOLFIRI/placebo according to age. In patients ≥ 65 years old OS median was 12.6 versus 11.3 months, in FOLFIRI/aflibercept and FOLFIRI/placebo, respectively. In those patients < 65 years old, OS median was 14.5 versus 12.5 months, in aflibercept and placebo treatment, respectively. Data demonstrated that patients < 65 and ≥ 65 years old benefit on both OS and PFS, hence the improvement in OS and PFS was associated with the addition of aflibercept to FOLFIRI compared with placebo. Safety FOLFIRI-Aflibercept is important, especially in elderly population. In this analysis, the incidence of AEs was higher for patients ≥ 65 years old than for those < 65 years old in both the aflibercept (89.3% vs 80.5%) and placebo (67.4% vs 59.4%) arms, and no heterogeneity between the older and younger patient populations was detected antiangiogenic agent-related AEs. Despite Interaction tests for grade 3/4 anti-VEGF-related did not suggest either evidence of heterogeneity (*p* > 0.1), the incidence of grade 3/4 anti-VEGF-related toxicities such as hypertension, venous thromboembolic events and proteinuria were, as expected, augmented by aflibercept. In this context, it should be mentioned that the number of patients with a treatment-emergent AE leading to permanent discontinuation was higher in the aflibercept plus FOLFIRI arm compared with those patients who were treated with placebo plus FOLFIRI (26.8% vs 12.1%, respectively). Regarding treatment-emergent AEs leading to treatment discontinuation, no analysis was conducted for compare patients aged < 65 years with those ≥ 65 years. [48]. Recently, OZONE trial evaluated the safety and effectiveness of aflibercept plus FOLFIRI in patients with mCRC treated in daily practice with a less selected patients’ population. not revealing major differences in the safety profile according to age in the subgroup analyses.

Occasionally, modified regimens are used to try to minimize the side effects of treatment. Thus, Ana Fernandez Montes et al. [49] reported that more than half of the patients in real world setting received modified FOLFIRI schedules or FOLFIRI at nonstandard dosages schedules from the very beginning (the study included 44% of the cases being aged more than 65 years versus 36% in the pivotal trial). All in all, survival‐related endpoints (median OS and PFS of 13.5 and 6.05 months, respectively) concur with those of the VELOUR study, 13.5 and 6.9 months [50].

The impact of the use of aflibercept on the quality of life of patients was also analyzed, with patients maintaining their quality of life throughout treatment [51].

Currently, different prospective studies with FOLFIRI-aflibercept exclusively in the elderly population are ongoing, which will confirm the efficacy and safety data reported with this treatment in this increasingly frequent population in our environment.

## Conclusion

According to current scientific evidence, Aflibercept is a really consistent and high evidence supported option, in combination with FOLFIRI, for those adult patients who are resistant to or has progressed after an oxaliplatin-containing regimen. Specifically, for RAS wild-type and RAS mutant patients, being aflibercept an alternative antiangiogenic recommended by ESMO guidelines for second-line treatment.

Treatment of RAS wt mCRC is a continuum of care, all lines of therapy should be planned upfront in order to achieve the best clinical benefit for the patient.

Aflibercept offers increased PFS, OS and RR in second line:

- The benefit of Aflibercept is independent of having received Bevacizumab in the 1st line, from the PFS to the first line and the RAS mutational status.
- In RAS wt mCRC patients, preclinical data and pooled clinical analysis support the use of first-line anti-EGFR and second-line anti-angiogenic. In real clinical practice, the efficacy of using aflibercept as a second-line treatment is maintained regardless of the previous use of anti-EGFR.
- There are not yet studies that assess the optimal sequence or the different antiangiogenics available in second line treatment nor predictive biomarkers.
- The second line treatment will be based on the patient's profile and the first line treatment received.
- The clinical benefit of using Aflibercept has been observed in different patients’ profiles: RAS mutant, RAS wild-type patients, and BRAF mutant patients, potentially resectable patients and elderly patients.
- Patients with rapid progression at first-line treatment can obtain clinical benefit after being treated with aflibercept.

Further, in our review with experts, they agree that specific mechanism of action confers aflibercept a total distinction from the rest of antiangiogenics, it has demonstrated to be an effective treatment.
